# A Mycovirus Representing a Novel Lineage and a Mitovirus of *Botrytis cinerea* Co-Infect a Basidiomycetous Fungus, *Schizophyllum commune*

**DOI:** 10.3390/v16111767

**Published:** 2024-11-13

**Authors:** Jie Duan, Anmeng Zhang, Yanping Fu, Yang Lin, Jiatao Xie, Jiasen Cheng, Tao Chen, Bo Li, Xiao Yu, Xueliang Lyu, Daohong Jiang

**Affiliations:** 1 State Key Laboratory of Agricultural Microbiology, Huazhong Agricultural University, Wuhan 430070, China; 18404968698@webmail.hzau.edu.cn (J.D.); 15071202759@163.com (A.Z.); jiataoxie@mail.hzau.edu.cn (J.X.); jiasencheng@mail.hzau.edu.cn (J.C.); taochen@mail.hzau.edu.cn (T.C.); boli@mail.hzau.edu.cn (B.L.); xiaoyu@mail.hzau.edu.cn (X.Y.); lvxueliang0715@webmail.hzau.edu.cn (X.L.); 2 The Provincial Key Lab of Plant Pathology of Hubei Province, College of Plant Science and Technology, Huazhong Agricultural University, Wuhan 430070, China; yanpingfu@mail.hzau.edu.cn (Y.F.); yanglin@mail.hzau.edu.cn (Y.L.)

**Keywords:** *Schizophyllum commune*, *Benyviridae*, *Mitoviridae*, *Botrytis cinerea*, mycovirus

## Abstract

Strain IBc-114 was isolated from a gray mold lesion and was identified as the fungus *Schizophyllum commune.* In this strain, two mycoviruses, Schizophyllum commune RNA virus 1 (ScRV1, C_AA053475.1) and Botrytis cinerea mitovirus 9 strain IBc-114 (BcMV9/IBc-114, C_AA053476.1), were isolated and characterized. ScRV1 has flexuous filamentous particles about 20 ± 2.1 nm in diameter and 1000 ± 94.2 nm in length. The genome of ScRV1 is 7370 nt in length and contains two open reading frames (ORFs) which encode a polyprotein and a coat protein, respectively. The polyprotein has 1967 aa, including a helicase domain and an RdRp domain which has the highest identity of 28.21% with that of Entomophthora benyvirus E (EbVE). The coat protein has 241 aa which is mostly phylogenetically close to the coat proteins of *Alphatetraviridae*. Based on the phylogenetic analysis of ScRV1 and viruses selected, ScRV1 might represent a new family (temporarily named Mycobenyviridae) of the order *Hepelivirales*. The genome of BcMV9/IBc-114 that infects *S. commune* is 2729 nt in length and has only one ORF encoding an RdRp protein with 719 aa. BcMV9/IBc-114 has the highest identity of 98.61% with Botrytis cinerea mitovirus 9 (BcMV9) (MT089704). ScRV1, but not BcMV9/IBc-114, has certain effects on the host growth of *S. commune*. Furthermore, BcMV9/IBc-114 has been demonstrated to replicate in the ascomycetous fungi *Botrytis cinerea* and *Sclerotinia sclerotiorum*, and it negatively affects the growth and pathogenicity of *B. cinerea*, but it does not affect *S. sclerotiorum*. This is the first report of mycoviruses in *S. commune* and cross-phyla transmission of mitovirus in nature.

## 1. Introduction

Mycoviruses, or fungal viruses, are viruses that infect fungi and oomycetes [1,2]. A variety of mycoviruses have been identified in fungi [3] with some of them reducing the growth rate or virulence of the host [4,5]. Mycoviruses have been assigned to 41 viral families and 1 viral genus by the International Committee on Taxonomy of Viruses (ICTV) (https://ictv.global/vmr/current, accessed on 6 August 2024). At present, mycoviruses are mainly reported and studied in plant pathogenic fungi since they can be used for the biocontrol of several fungal diseases of the plants [6]. The *Schizophyllum commune* belongs to the family Schizophyllaceae in the order Agaricales of the Phylum Basidiomycota and is widely distributed all over the world [7]. It is an important edible and medicinal fungus. It has a high cultural value in the mushroom-selling market and has anti-breast cancer activity [8,9]. Despite the wide distribution of *S. commune*, studies on mycoviruses infecting this species are scarce. Only one spherical virus-like particle, measuring 130 nm in diameter, has been previously observed [10].

*Botrytis cinerea*, an ascomycetous fungus, is a cosmopolitan plant pathogen that can infect more than 3000 plant species (https://nt.ars-grin.gov/fungaldatabases/, accessed on 6 August 2024), and mycoviruses that infect *B. cinerea* have been thoroughly investigated [1,3]. Certain mycoviruses can naturally occur in fungi that belong to phylogenetically distant species [11,12,13,14,15,16,17]. Additionally, some mycoviruses can be artificially transmitted between these phylogenetically distant fungal species [18,19,20]. The above results indicated that the cross-species transmission of mycoviruses may occur in nature. The cross-species transmission of mycoviruses will expand the host range of mycoviruses, making it more conducive to the survival of mycoviruses in nature. However, the mechanism of cross-species transmission of mycoviruses is still unknown at present.

In this study, a strain IBc-114 collected from a gray mold lesion on cucumber in Achituv, Israel, initially was firstly regarded as an abnormal strain of *B. cinerea*; while after carefully identifying by using DNA sequences of ITS and other selected genes, it was identified as *S. commune* [21]. Previously, virome data were obtained by sequencing the RNA extracted from Israel strains of *B. cinerea*, including strain IBc-114. Two mycoviruses were successfully detected in strain IBc-114 by RT-PCR: one is a novel beny-like virus, and another is a mitovirus which has been identified in *B. cinerea*. In this study, the viral particles and genome characteristics of these two mycoviruses and the cross-phyla transmission of the mitovirus were investigated, and a novel virus family was proposed based on the phylogenetic analysis.

## 2. Materials and Methods

### 2.1. Fungal Isolates and Culture Conditions

*S. commune* strain IBc-114 was collected from a gray mold lesion on cucumber in Achituv, Israel, on 14 January 2019. Both *S. sclerotiorum* strain Ep-1PNA367 and *B. cinerea* strain B05.10 were previously preserved in our laboratory. The specific information of the two strains is the same as that of the strains used by Zhang et al. previously [22]. Three strains were incubated on potato dextrose agar (PDA) [23] at 20 °C. The recipe for PDA medium is as follows: potato extract (from boiling 200 g of potatoes in 1 L of water), 20 g/L glucose, and 20 g/L agar. Stock cultures of IBc-114 were maintained on PDA and stored at 4 °C.

### 2.2. Identification of Strain IBc-114

Hyphal agar plugs of strain IBc-114 were inoculated on Petri dishes (9 cm diam.) containing PDA overlaid with sterilized cellophane and incubated at 20 °C in the dark for 5 days. Hyphae were harvested using a sterilized spoon and transferred to 2.0 mL centrifuge tubes. The extraction of genomic DNA was conducted using the cetylramethylammonium bromide (CTAB) method. The concentration and quality of DNA were detected by a NanoDrop 2000 Spectrophotometer (Wilmington, DE, USA) and agarose gel electrophoresis, and the extracted DNA was dissolved in RNase water and stored at −20 °C until use. The extracted DNA dissolved in RNase water was used as a template for the amplification of an internal transcribed spacer (ITS), *Calmodulin*, guanosine triphosphate (GTP)-binding protein gene (*Ras*) and *β-tubulin* [21] using the specific primers shown in Table S1. The composition of the PCR mix is as follows: 2 × Hieff^®^ Robust PCR Master Mix (With Dye) (Yeasen Biotech Co., Ltd., Shanghai, China): 12.5 μL; forward primer (0.2 μM): 0.5 μL; reverse primer (0.2 μM): 0.5 μL; template DNA (50 ng/μL, genomic DNA): 1 μL; and we added deionized water to make the total volume up to 25 μL. The amplification procedure is as follows: pre-denaturation at 95 °C for 5 min; denaturation at 95 °C for 30 s; annealing at 56 °C for 30 s; extension at 72 °C for 30 s; repeat from the second step for 34 cycles; then perform a final extension at 72 °C for 5 min; store at 16 °C for 2 min. Detect the target fragment by 1% agarose gel electrophoresis. The amplified product was cloned into a pMD™ 19-T Vector Cloning Kit (Takara, Dalian, China) and sequenced. Subsequently, sequences were analyzed to search for homologies with fungal sequences using BLASTn in the NCBI database. A phylogenetic tree based on ITS, *Calmodulin*, *Ras* and *β-tubulin* sequences of selected fungi was constructed by the maximum likelihood (ML) method using IQ-Tree [24] with 1000 bootstrap replications (Table S2).

### 2.3. Total RNA and dsRNA Extraction, Sequencing and Detection of Mycoviruses in Strain IBc-114

The total RNA of strain IBc-114 was extracted by the Trizol method [25]. The concentration and quality of RNA were measured by a NanoDrop 2000 Spectrophotometer (Wilmington, DE, USA) and agarose gel electrophoresis, and then the RNA sample was stored at −80 °C. The dsRNA was extracted as previously described [26] and followed by purification to remove DNA and ssRNA by digestion with S1 nuclease and DNase I (TaKaRa, Dalian, China) [26]. The dsRNA samples were used for mycovirus detection and mycoviral terminal sequence determination.

RNA-Seq was performed on a mixed total RNA sample of strains containing strain IBc-114 by GENEWIZ Technology Services (Suzhou, China). After sequencing, quality control, assembly and other processes were performed on the raw reads as previously described [27]. Eventually, viral contigs were generated. The first-strand cDNA was synthesized with random primer by using cDNA Synthesis SuperMix (TransGen Biotech, Beijing, China). It was carried out as follows: the mixture of RNA dissolved in RNase-free water and random primers was firstly heated at 65 °C for 5 min and chilled on ice for 5 min; then, the reverse transcription (RT) reaction was continued at 25 °C for 10 min, 42 °C for 30 min, and 85 °C for 15 s. And the PCR reaction was as follows: pre-denaturation at 95 °C for 5 min; denaturation at 95 °C for 30 s; annealing at 56 °C for 30 s; extension at 72 °C for 60 s; repeat from the second step for 34 cycles; then perform a final extension at 72 °C for 5 min; store at 16 °C for 2 min.

Viruses were detected by RT-PCR using virus-specific primers designed based on the assembled mycovirus contig sequences. All primers are listed in Table S1.

### 2.4. Cloning of Full-Length Genome Sequence of Mycoviruses

The terminal sequence of all mycoviruses were obtained by referring to Potgieter et al.’s amplification method [28]. The dsRNA (200–500 ng) was mixed with 30 pmol of primer PC3-T7 (5′-p-GGATCCCGGGAATTCGGTAATACGACTCACTATATTTTTATAGTGAGTCGTAT TA-OH-3′), and the PC3-T7 loop was ligated to the 3′-terminal of each viral RNA strand using T4 RNA ligase (40 U/μL, TaKaRa, Dalian, China) at 16 °C for 12 h. The adaptor-ligated viral RNAs were reverse transcribed using M-MuLV reverse transcriptase (200 U/μL, TaKaRa, Dalian, China). The synthesized cDNA (1 μL) was subsequently used for PCR amplification using the primer PC2 (5′-CCGAATTCCCGGGATCC-3′), which is complementary to the PC3-T7 loop, and sequence-specific primers corresponding to the 5′- or 3′-terminal sequence of the contigs. The amplified products were cloned into the vector pMD-19T and sequenced. The full-length genome sequence of the putative viruses was assembled based on the sequencing results.

### 2.5. Analysis of Mycoviral Genome Sequences

An assembly of mycoviral sequences and prediction of mycoviral open reading frames (ORFs) were performed using DNAMAN. The mycoviral sequences were downloaded from the NCBI database. Multiple mycoviral sequences were aligned using MAFFT (Version 7.520). The phylogenetic analysis was conducted via maximum likelihood (ML) methods with 1000 replicates using IQ-TREE (Version 2.2.2.7). The bootstrap values (>50%) are labeled on branches. The sequences of the selected mycoviral isolates used for phylogenetic analysis were downloaded from NCBI.

### 2.6. Virus Particle Extraction and Observation

The viral particles were isolated from strain IBc-114 by referring to the method described by Liu et al. [26]. The strain was grown at 20 °C for 10 d on sterilized cellophane films placed on PDA. Approximately 50 g of mycelia was ground in liquid nitrogen and mixed with five volumes of 0.05 M phosphate buffer (pH 7.4) containing 0.05% mercaptoethanol (*v*/*v*), and then the mixture was gently shaken on ice for 1 h. The mixture was separated by using a gradient centrifugation (at 3500 r/min, 7000 r/min, and <10,000 r/min for 10 min); then, the supernatant was collected and ultracentrifugation (30,000 r/min for 4 h) was performed. Finally, 1 mL of 0.05 M PBS (pH 7.4) was added to dissolve the precipitate to obtain the crude extract. The crude extract was centrifuged at 30,000 r/min for 4 h through 10–60% sucrose gradient centrifugation, and the virus particles were distributed at 30–40%. After careful collection, the solution was dissolved in 200 μL of 0.05 M PBS. The virus particles were stained with 2% (*w*/*v*) phosphotungstic acid (PTA) solution (pH 7.4) and observed using a transmission electron microscope (TEM; Model Tecnai G2, 200 kV, FEI Company, Hillsboro, OR, USA).

### 2.7. 4D-FastDIA-Based Proteomic of Proteins in 40% Sucrose Solution

Total proteins from 40% sucrose solution were denatured in boiling water. The denatured proteins were then subjected to 4D-FastDIA by PTM Biolabs Inc., Hangzhou, China. The database obtained was subsequently utilized to search for peptides associated with ScRV1 and BcMV9/IBc-114.

### 2.8. Protoplast Preparation and Biological Characteristics of Virus-Infected Strains

Mycelia of three strains (IB-114, B05.10 and Ep-1PNA367) were incubated overnight in PDB for 20 h. Protoplasts were prepared by incubating the hyphae in a solution of 0.3% *w*/*v* megalyase solution in 0.7 M NaCl for 2 h at 28 °C on a shaker at 100 rpm. The mixture was filtered through a single layer of filter paper and then centrifuged at 6000 rpm for 10 min; the resulting protoplast pellet was washed twice with 0.7 M NaCl and resuspended in 0.5 mL of STC buffer (1 M sorbitol, 50 mM Tris pH 8.0, and 50 mM CaCl_2_·2H_2_O). The protoplast suspension of strain IBc-114 was diluted 50 times, 100 times, 300 times, 500 times, and 1000 times, respectively. Then, the suspensions were spread on regeneration medium (1 g/L casein hydrolysate, 1 g/L yeast extract, 239.4 g/L sucrose, 16 g/L agar) containing 50 mg/mL cefotaxime and 100 mg/mL ribavirin and incubated at 20 °C for 7 to 8 days. Single colonies were picked and cultured on PDA plates, and RT-PCR was used to detect whether strain IBc-114 was successfully freed from virus infection. The protoplast suspension of two strains (B05.10 and Ep-1PNA367) was spread onto regeneration medium and incubated at 20 °C for 7 to 8 days. More than 10 mycelial plugs were cut from the regenerated colonies with a cork borer at random and transferred onto fresh PDA plates. The total RNA of the isolates was extracted and were used for virus detection with RT-PCR.

In order to investigate the effects of mycoviruses, virus-free protoplast progenies (IBc-114-39 and IBc-114-46, which are the protoplast progenies of IBc-114) were obtained. To determine the growth rate, strains IBc-114, IBc-114-39 and IBc-114-46 were inoculated on 20 mL PDA medium and incubated at 20 °C. The colony diameter was recorded at 24 and 36 h post-inoculation (hpi). The growth rate of each strain was calculated, and the colony morphology on PDA was photographed at 14 days post-inoculation (dpi).

### 2.9. Transfection with the Viral RNAs

The total RNA (1 μg) of IBc-114 was added to 100 µL of protoplasts (about 1 × 10^8^/mL) (Strains B05.10 and Ep-1PNA367) for a transfection assay; PEG-mediated transfection was conducted according to previous reports [29]. Transfected protoplasts were mixed with regeneration medium at 48 °C and incubated for 5 to 10 days at 20 °C. The colonies were transferred onto a fresh PDA plate overlaid with cellophane and incubated for 2 days at 20 °C. The total RNA of the strains was extracted and used for virus detection with RT-PCR.

### 2.10. Phenotypic Characteristics and Virulence Assay of Transfectants

To assess the growth characteristics of *B. cinerea* and *S. sclerotiorum* transfectants in this study, agar plugs of fresh mycelium were transferred from colony margins of old cultures onto fresh PDA medium. The colony radius of each strain was measured daily for 3 days, and the growth rate of each strain was calculated. To evaluate the virulence of the fungal strain, freshly grown mycelial agar plugs were inoculated onto the detached tomato fruits and incubated at 20 °C with 90% humidity. There were five replicates for each control (Strains B05.10 and Ep-1PNA367) and treatment (Strains B05.10-P1-114 and Ep-1PNA367-P5-114).

### 2.11. Viral Horizontal Transmission and Detection of Mycoviruses by RT-PCR

Horizontal transmission refers to the transmission of mycoviruses from a fungal strain to other fungal strains through hyphal contact or anastomosis [30]. In each pairing culture (9 cm in the diameter of the petri dish), two strains, i.e., one donor strain, IBc-114, and one recipient strain, IBc-114-39, were used in this experiment, using the pairing culture technique described by Hao et al. [31]. Derivative isolates were obtained from the recipient strain IBc-114-39 in the contact cultures using the method described by Wu et al. [32]. All derivative isolates were subjected to test for the presence of *Actin* gene and two mycoviruses through RT-PCR with primer pairs SC-ACTIN-F1/SC-ACTIN-R1, ScRV1-F3/ScRV1-R3 and ScMV1-F/ScMV1-R, respectively (Table S1). Strains IBc-114 and IBc-114-39 were included as controls in this experiment. Six derivative isolates, namely R1, R2, R3, R4, R5 and R6, were selected and individually tested for mycelial growth rate and the colony morphologies on PDA (20 °C).

### 2.12. Data Analyses

Experimental data were subjected to analysis of variance by using the software SPSS (version 10.0). Treatment means were compared by using the least-significant-difference test at a significance level of a *p* value of 0.05. The growth rate of strains is calculated by the cross method [33]. The calculation of the lesion area of detached tomato fruits was carried out using Image J software (version 1.8.0).

### 2.13. Viral and Nucleotide Sequence Accession Numbers

The data reported in this paper have been deposited in the GenBase [34] in National Genomics Data Center [35], Beijing Institute of Genomics, Chinese Academy of Sciences/China National Center for Bioinformation, under accession number C_AA053475.1, C_AA053476.1, C_AA098060.1, C_AA098061.1, C_AA098062.1 and C_AA098063.1 that is publicly accessible at https://ngdc.cncb.ac.cn/genbase, accessed on 2 November 2024.

## 3. Results

### 3.1. Identification of Strain IBc-114 as Schizophyllum commune

**Figure 1:**
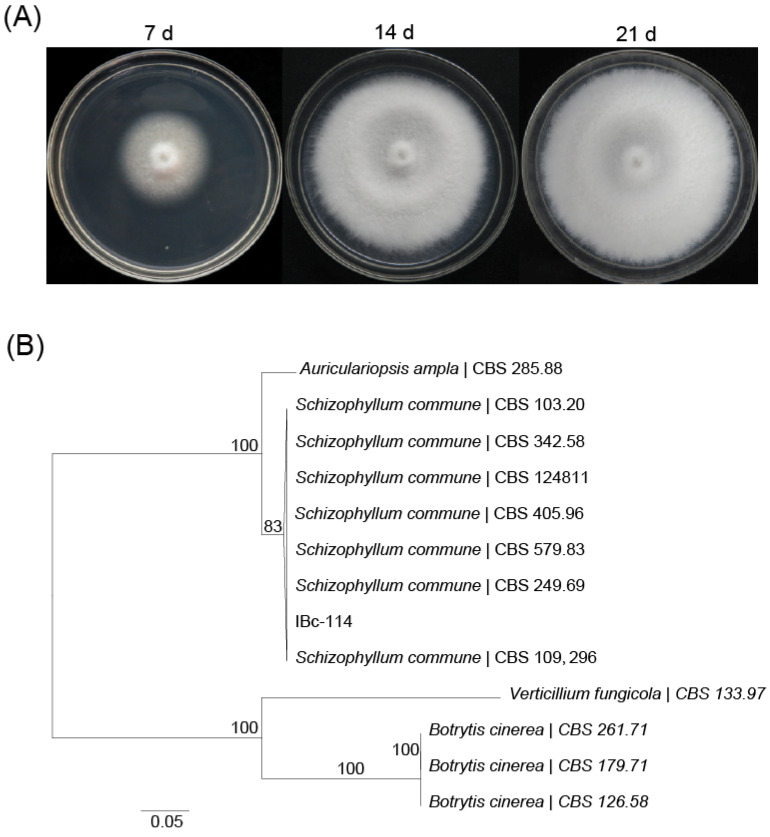
Identification of strain IBc-114. (**A**) Colony morphology of strain IBc-114. All strains were cultured on plates with 20 mL of PDA medium at 20 °C. (**B**) Maximum-likelihood tree inferred from ITS, *Calmodulin*, *Ras* and *β-tubulin* sequences. Numbers above nodes indicate bootstrap percentage per 1000 replicates. Fungal species and associated CBS strains are listed in Table S2.

Strain IBc-114, a conidiation defective fungus, was originally isolated from a gray mold lesion on cucumbers in Israel, and it was regarded as an abnormal strain of *B. cinerea*. This strain was further identified based on its morphological features and molecular characters by sequences of ITS, *Calmodulin*, *Ras*, and *β-tubulin*. A colony with white, densely woolly mycelia was observed on PDA after 14 d of incubation at 20 °C, and the fungal isolate produced a characteristic tart and unpleasant smell. The DNA sequences of ITS, *Calmodulin*, *Ras* and *β-tubulin* of the strain IBc-114 were 99.53%, 93.45%, 97.27% and 95.94% identical to those of *S. commune*, respectively. The multigene phylogenetic analysis indicated further that strain IBc-114 was *S. commune* (Figure 1B). Thus, IBc-114 was identified as a strain of *S. commune.*

### 3.2. Identification of Two Mycoviruses in Strain IBc-114

**Figure 2:**
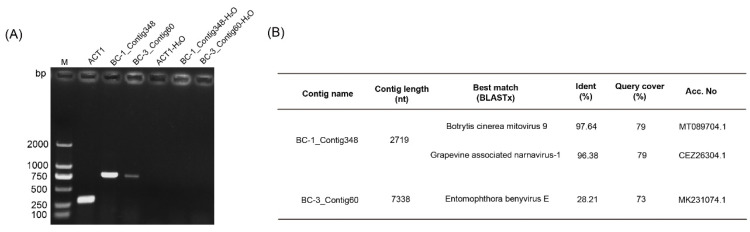
Detection and information of viral contigs in strain IBc-114 using RT-PCR. (**A**) Detection of viral contigs in strain IBc-114 using RT-PCR. Lane M: DL2000, DNA marker; ACT1: *S. commune* actin 1 gene (442 bp); BC-1_Contig348: 801 bp; BC-3_Contig60: 798 bp; ACT1-H_2_O, BC-1_Contig348-H_2_O and BC-3_Contig60-H_2_O: H_2_O was used as a negative control. (**B**) Information of mycoviruses obtained from high-throughput sequencing (HTS) analysis of the *Schizophyllum commune* strain IBc-114.

The RNA library sequenced included pooled RNA from 71 strains, 70 *B. cinerea* and one *S. commune* (IBc-114). To detect the viruses in IBc-114, primer pairs designed based on the sequence of each viral contig were used to examine the RNA sample of IBc-114. Target RT-PCR products could be amplified by two primer pairs designed on viral BC-1_Contig348 and BC-3_Contig60 (Figure 2). BC-1_Contig348 was 2719 nt long with one large complete ORF that was predicted to encode an RdRp with 718 amino acids. Based on BLASTx analysis, the RdRp showed 97.64% and 96.38% identity with those of Botrytis cinerea mitovirus 9 (BcMV9) [16,36]. BC-3_Contig60 was 7338 nt long with one large complete ORF and one small ORF. Based on BLASTx analysis, the large ORF showed 28.21% identity with the polyprotein of Entomophthora benyvirus E; the small ORF shared no identity with any proteins in the NCBI database. Thus, IBc-114 is co-infected by two mycoviruses: one is BcMV9 and another is relatively close to the family *Benyviridae*; these two viruses were identified as Botrytis cinerea mitovirus 9 strain IBc-114 (BcMV9/IBc-114) and Schizophyllum commune RNA virus 1 (ScRV1), respectively.

### 3.3. Schizophyllum commune RNA Virus 1 (ScRV1)

#### 3.3.1. Complete Sequence and Organization of ScRV1 Genome

**Figure 3:**
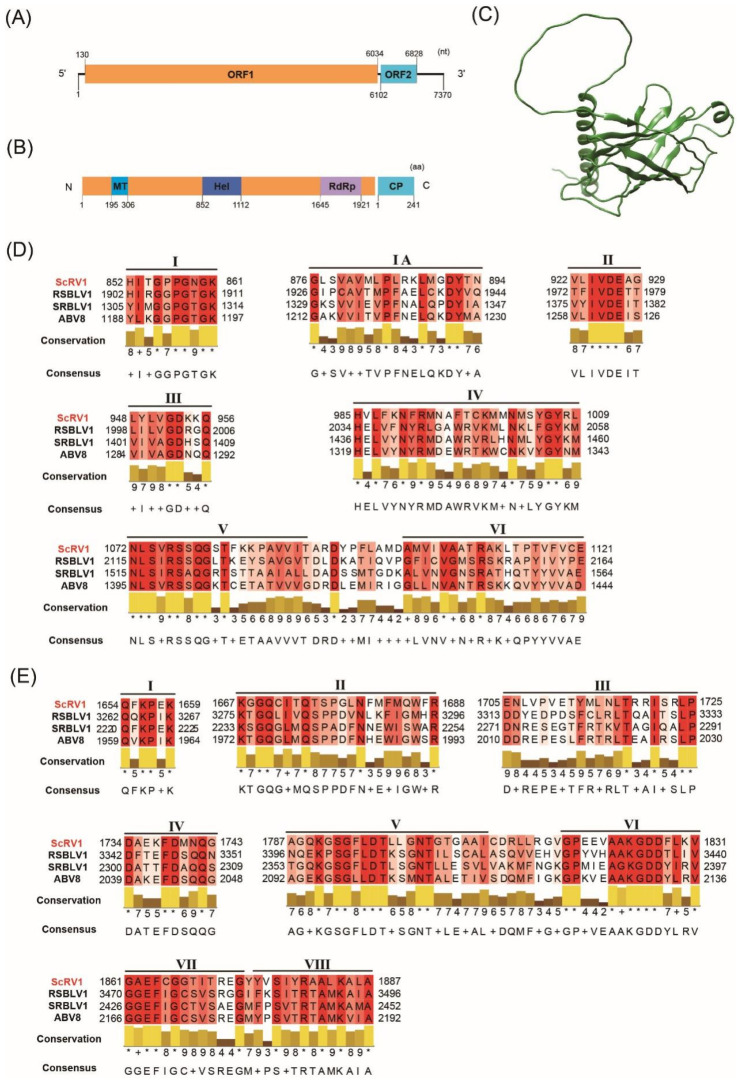
Diagrammatic sketch of the genome organization of ScRV1 and the alignment of RdRp domain and helicase (Hel) domains of ScRV1 with beny-like viruses. (**A**) The genome organization of SsBLV1, showing 5′ and 3′ untranslated regions and two ORF regions. (**B**) The polyprotein encoded by ORF1 of ScRV1 and conserved domains Hel and RdRp and the coat protein (CP) encoded by ORF2. The Arabic numerals show the amino acid position of each conserved domain. (**C**) The structure of the CP protein of ScRV1 obtained by prediction using AlphaFold2. (**D**) Multiple alignment of ScRV1 Hel motifs with those of selected beny-like viruses. Conserved motifs are marked by Roman numerals from I to VI. (**E**) Multiple alignment of ScRV1 RdRp motifs with those of selected beny-like viruses. Conserved motifs are marked by Roman numerals from I to VIII. Identical residues are indicated by asterisks and highlighted in black.

The full-length genome of ScRV1 contains a single RNA segment consisting of 7370 nt. ScRV1 has a 5′ untranslated region (UTR) of 129 nt and a 3′ UTR of 542 nt, and its GC content is 54.1% (Figure 3A). Sequence analysis revealed that ScRV1 has a large ORF1 (nt positions 130 to 6034) encoding a polyprotein of 1967 amino acid residues and a small ORF2 (nt positions 6102 to 6828) encoding a protein of 241 amino acid residues (Figure 3A). The polyprotein was predicted to contain a viral methyltransferase (MT) from 195 to 306 aa, a viral RNA helicase (Hel) from 852 to 1112 aa and a viral RdRp from 1645 to 1921 aa (Figure 3B). The Hel and RdRp of ScRV1 shared six typical conserved motifs (I–VI) and eight typical conserved motifs (I–VIII) with other beny-like viruses, respectively (Figure 3D,E). BLASTp analysis demonstrated that the Hel and RdRp domains of ScRV1 have the highest identity of 32.74% and 40.42% with those of Entomophthora benyvirus E (EbVE) (QED42962), respectively. There is no significant similarity between the protein encoded by ORF2 and the known proteins in NCBI. The results of homology analysis of the hidden Markov model (HMM) of the protein showed that it shares high homology with the capsid protein of the Hepatitis E virus, Avian hepatitis E virus, Beet necrotic yellow vein virus and Prunus necrotic ringspot virus (Table 1); thus, it might be a putative CP protein. The structure of the CP protein was further predicted by AlphaFold2 (Figure 3C).

#### 3.3.2. Purification of Viral Particles from Strain IBc-114

**Figure 4:**
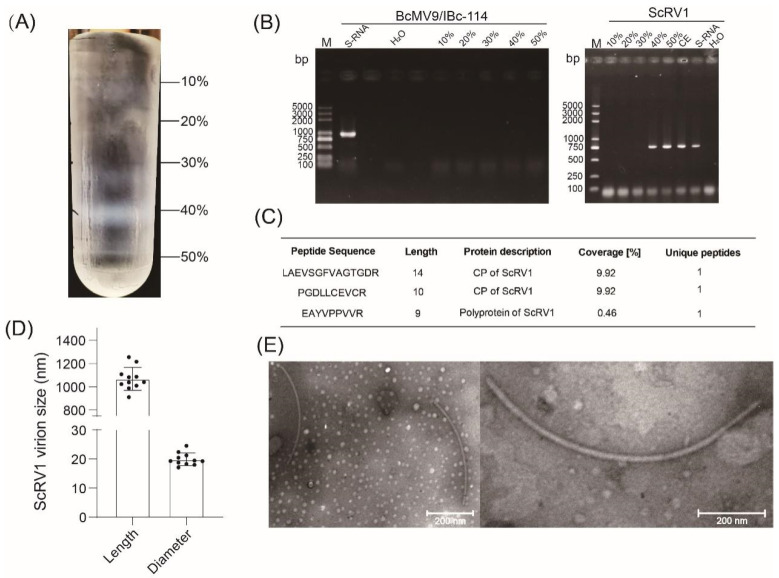
Identification of virions from strain IBc-114. (**A**) The distribution of protein in the crude extract of virions was analyzed in a 10–60% sucrose gradient solution after ultracentrifugation. (**B**) RT-PCR detection of BcMV9/IBc-114 and ScRV1 in different sucrose concentration gradients. Lane M: DL5000, DNA marker; S-RNA: RT-PCR detection of BcMV9/IBc-114 in strain IBc-114; 10%, 20%, 30%, 40%, 50%, 60%: RT-PCR was performed on BcMV9/IBc-114 and ScRV1 in 10–60% sucrose solutions after ultracentrifugation, respectively; CE: ScRV1 in crude extract containing virions was detected by RT-PCR; H_2_O: ddH_2_O was used instead of RT products. (**C**) CP and RdRp peptide information of ScRV1 in 40% sucrose solution determined by 4DFastDIA-based proteomic. (**D**) A histogram of length and diameter of ScRV1 particles. Error bars indicate standard deviation (SD) from sample means. (**E**) Virions of ScRV1 observed under a TEM after negative staining.

To determine whether the viruses infecting strain IBc-114 were encapsidated, virus particles were purified via ultracentrifugation. After sucrose gradient centrifugation, distinct protein bands can be observed in 10%, 30%, 40%, and 50% sucrose solutions (Figure 4A). After being desucrosed, BcMV9/IBc-230 and ScRV1 were analyzed using RT-PCR. The RT-PCR results indicated that BcMV9 could not be detected in any of the sucrose gradients, whereas ScRV1 was detectable in the 40% and 50% sucrose gradients (Figure 4B). The results of 4D-FastDIA also showed that the peptides of ScRV1 could be detected in a 40% sucrose gradient, while the peptide of BcMV9 was not detected (Figure 4C). The viral particles isolated from strain IBc-114 were negatively stained by phosphotungstic-acid solution and examined by transmission electron microscopy (TEM). The results revealed the presence of flexuous filamentous particles with 20 ± 2.1 nm in diameter and 1000 ± 94.2 nm in length (Figure 4D,E). The above results showed that virions extracted from strain IBc-114 belonged to ScRV1.

#### 3.3.3. Phylogenetic Analysis of ScRV1

**Figure 5:**
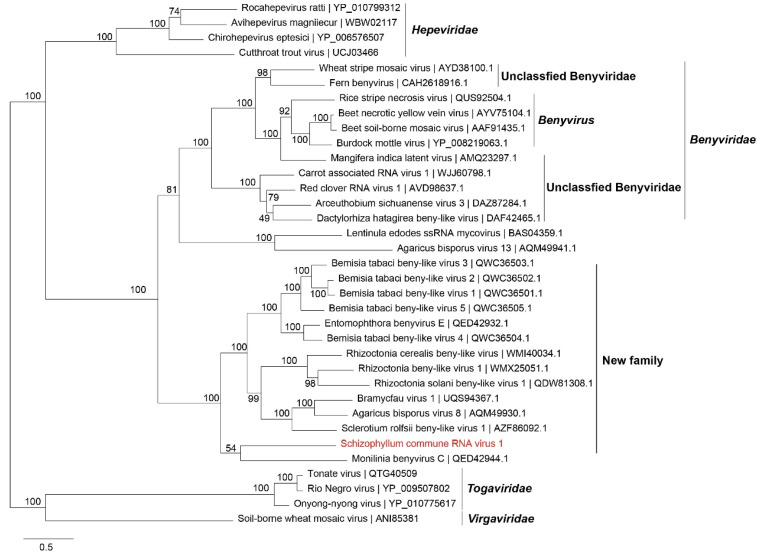
Phylogenetic analysis of ScRV1 based on the polyprotein using maximum likelihood with 1000 bootstrap replicates. Phylogenetic tree of ScRV1 based on the polyproteins constructed with the best-fit model Q.pfam + F + R5. The parameter of bootstrap was set as 1000 replicates, and bootstrap values over 50% are indicated on branches. The virus reported in this study is in red; the scale bar at the bottom left corresponds to the genetic distance.

**Figure 6:**
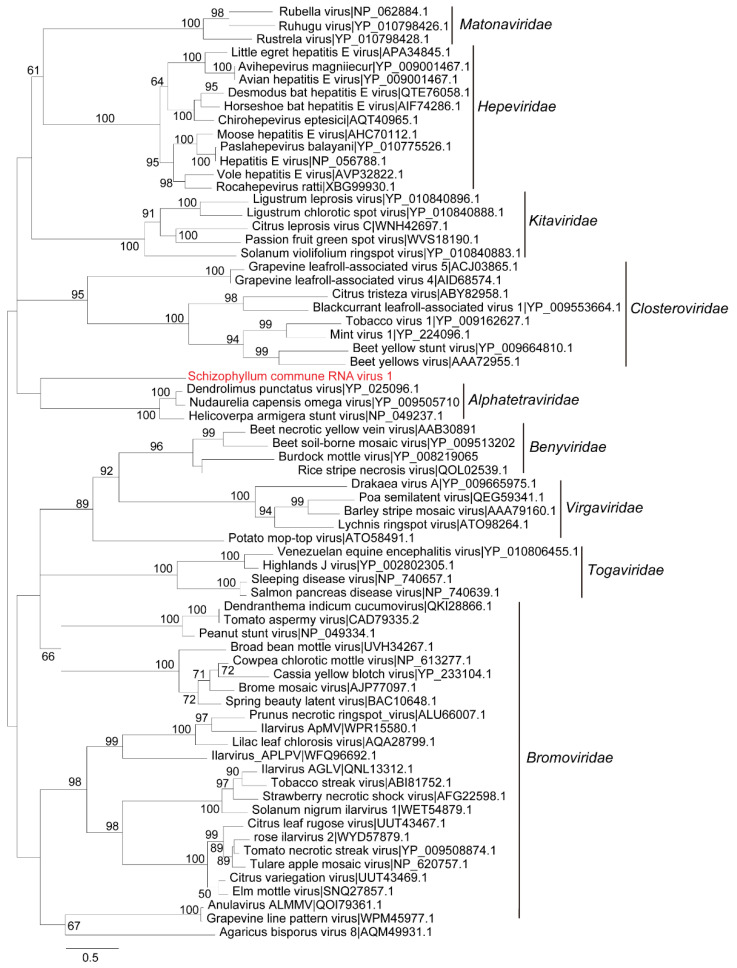
Phylogenetic analysis of ScRV1 based on the CP using maximum likelihood with 1000 bootstrap replicates. Phylogenetic tree of ScRV1 based on the CPs constructed with the best-fit model Q.pfam + F + I + G4. The parameter of bootstrap was set as 1000 replicates, and bootstrap values over 50% were indicated on branches. The virus reported in this study is in red; the scale bar at the bottom left corresponds to the genetic distance.

A polyprotein sequence of ScRV1 was used on the BLAST/NCBI database. Then, protein sequences of benyviruses and other related viruses with complete polyproteins were downloaded and used to perform phylogenetic analysis by the maximum-likelihood method with IQ-Tree. The phylogenetic tree showed that ScRV1 and the previously reported beny-like viruses in *Sclerotium rolfsii*, *Agaricus bisporus*, *Rhizoctonia solani*, *Bemisia tabaci*, *Monilinia*, and *Entomophthora* form a distinct evolutionary branch independent from the branch of *Benyviridae* family (Figure 5). In addition, the CP protein of ScRV1 is evolutionarily closely related to members of *Alphatetraviridae* but not related to those of benyviruses (Figure 6). Therefore, a new family, Mycobenyviridae, is proposed to accommodate these beny-like viruses found in fungi.

### 3.4. Genome of Botrytis cinerea Mitovirus 9 Strain IBc-114 (BcMV9/IBc-114)

**Figure 7:**
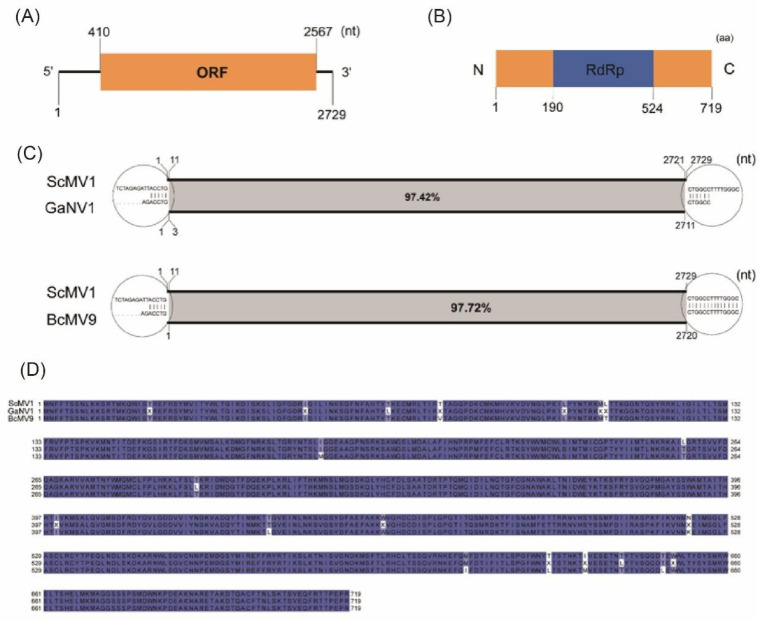
Diagrammatic sketch of the genome organization of BcMV9/IBc-114 and sequence alignment of BcMV9/IBc-114, GaNV1 and BcMV9. (**A**) The genome organization of BcMV9/IBc-114, showing 5′ and 3′ UTR and ORF regions. (**B**) The RdRp encoded by the ORF of BcMV9/IBc-114 and conserved RdRp domain. Arabic numerals show the amino acid position of the conserved domain. (**C**) Nucleotide sequence alignment of BcMV9/IBc-114, GaNV1 and BcMV9. (**D**) Amino acid sequence alignment of BcMV9/IBc-114, GaNV1 and BcMV9.

The genome of BcMV9/IBc-114 found in this study is 2729 nucleotides (nt) in length and has one ORF, which encodes an RdRp with 719 amino acid residues (Figure 7A,B). BLASTn analysis demonstrated that the genome of BcMV9/IBc-114 shares the highest identity of 97.79% and 97.53% with Botrytis cinerea mitovirus 9 (BcMV9, MT089704.1, 2720 nt) and Grapevine-associated narnavirus-1 (GaNV1, ON738339, 2711 nt), respectively (Figure 7C). The 5′ UTR of BcMV9/IBc-114 was 8 nucleotides longer than that of BcMV9, while the 3′ UTRs of the two viruses were identical (Figure 7C). The 5′ and 3′ UTRs of BcMV9/IBc-114 were 7 nt and 8 nt longer than those of GaNV1, respectively (Figure 7C). The RdRp values of BcMV9/IBc-114, BcMV9, and GaNV1 are all 719 amino acids in length. However, RdRp shares the highest identity of 97.77% and 98.61% with those of BcMV9 (QKW91256.1) and GaNV1 (CEZ26304.1), respectively (Figure 7D).

### 3.5. The Impact of Viruses ScRV1 and BcMV9/IBc-114 on the Host

**Figure 8:**
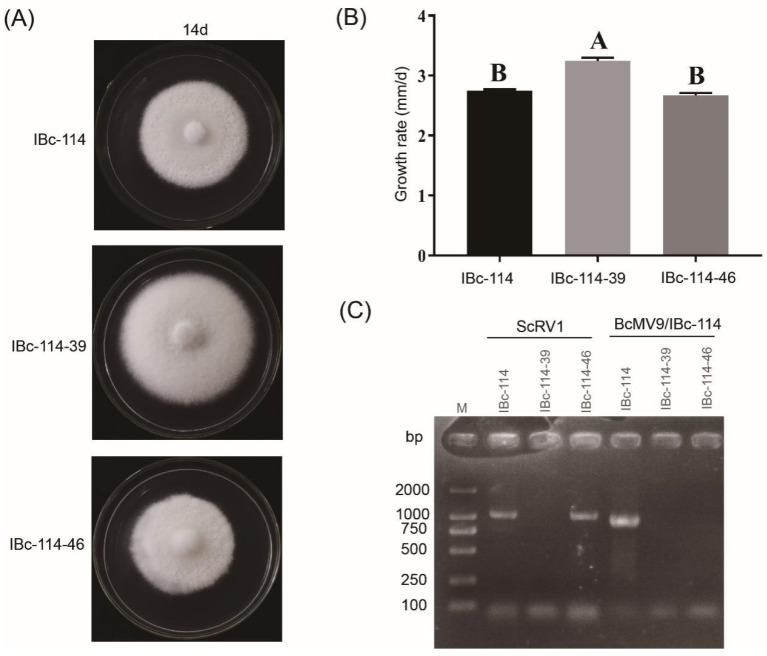
The biological characteristics of strains IBc-114, IBc-114-39, and IBc-114-46 and the virus detection using RT-PCR. (**A**) The colony morphology of strains IBc-114, IBc-114-39, and IBc-114-46 on PDA at 20 °C. (**B**) The growth rate of strains IBc-114, IBc-114-39, and IBc-114-46 on PDA at 20 °C. The growth rate was measured by recording the colony diameter at 24 h and 36 h. Error bars indicate standard deviation (SD) from sample means. Different uppercase letters on the top of each column indicate significant differences (*p* < 0.05). (**C**) Detection of viruses in strains IBc-114, IBc-114-39, and IBc-114-46. Lane M: DL2000, DNA marker.

Strains IBc-114-39 and IBc-114-46 were protoplast progenies of IBc-114. Strain IBc-114 was co-infected by ScRV1 and BcMV9/IBc-114, strain IBc-114-39 was not infected by any virus, and strain IBc-114-46 was only infected by ScRV1 (Figure 8A). The growth rate of the strains IBc-114, IBc-114-39 and IBc-114-46 on PDA at 20 °C was 2.75 mm/d, 3.25 mm/d and 2.67 mm/d, respectively, and the growth rate of strain IBc-114-39 was significantly higher than those of strains IBc-114 and IBc-114-46 (Figure 8B). There was no significant difference in morphological features among the three strains (Figure 8C). The results suggested that ScRV1 may adversely affect the host growth, while BcMV9/IBc-114 has no effects on the growth of the host.

### 3.6. Horizontal Transmission of ScRV1 and BcMV9/IBc-114

**Figure 9:**
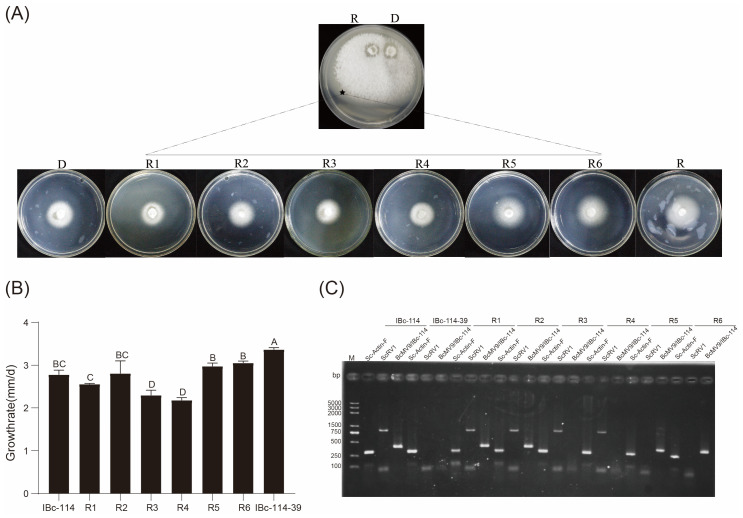
Horizontal transmission of ScRV1 and BcMV9/IBc-114, the donor strain IBc-114 (D, donor; ScRV1^+^, BcMV9/IBc-114^+^), to the recipient strain IBc-114-39 (R, recipient; ScRV1^−^, BcMV9/IBc-114^−^) through hyphal contact in a pair culture. (**A**) A pair culture of R/D on PDA (20 °C, 12 days), two single cultures of isolates R and D, respectively, and six derivative isolates (R1, R2, R3, R4, R5 and R6) on PDA (20 °C, 5 days). “★” in the pair culture indicates the area where a mycelial agar plug was removed for generating a derivative of the recipient strain designated as R. (**B**) Average growth rates (*n* = 3) for IBc-114 and IBc-114-39, six derivative isolates. Different letters on the bars in each graph indicate significant difference (*p* < 0.05) according to the least significant difference test. (**C**) Detection of *Actin* genes, ScRV1 and BcMV9/IBc-114 in IBc-114 and IBc-114-39, six derivative isolates by total RNA extraction and RT-PCR.

To determine the transmission capacity of ScRV1 and BcMV9/IBc-114, strain IBc-114 was dually cultured with strain IBc-114-39 on the same PDA plate for 12 days (Figure 9A). Six mycelial plugs were picked out from the positions indicated by the black stars shown in Figure 9A. The presence of two mycoviruses, ScRV1 and BcMV9/IBc-114, in all six derivative isolates was detected through reverse transcription (RT)-PCR with the specific primers (Table S1). The results showed that R1 and R2 contained two viruses, ScRV1 and BcMV9/IBc-114, R3 and R4 contained one virus, ScRV1, and R5 and R6 contained one virus, BcMV9/IBc-114 (Figure 9C). Therefore, ScRV1 and BcMV9/IBc-114 in strain IBc-114 can be horizontally transmitted to strain IBc-114-39 through hyphal contact.

Isolates R3 and R4 grew slower than two strains (IBc-114, IBc-114-39) or isolates (R1, R2, R3 and R4) with the average radial growth rate of 2.30 mm/day and 2.18 mm/day, respectively. Four isolates (R1, R2, R5 and R6) grew on PDA plates with average radial growth rates of 2.56 mm/day, 2.80 mm/day, 2.97 mm/day and 3.05 mm/day, respectively, and were comparable to strain IBc-114 with a radial growth rate of 2.78 mm/day. However, their growth rates were slower than that of strain IBc-114-39 with a radial growth rate of 3.37 mm/day (Figure 9B). These results further imply that ScRV1 has a negative effect on host growth. Meanwhile, BcMV9/IBc-114 has no impact on the growth of the host.

### 3.7. BcMV9/IBc-114 Can Successfully Infect B. cinerea and S. sclerotiorum

**Figure 10:**
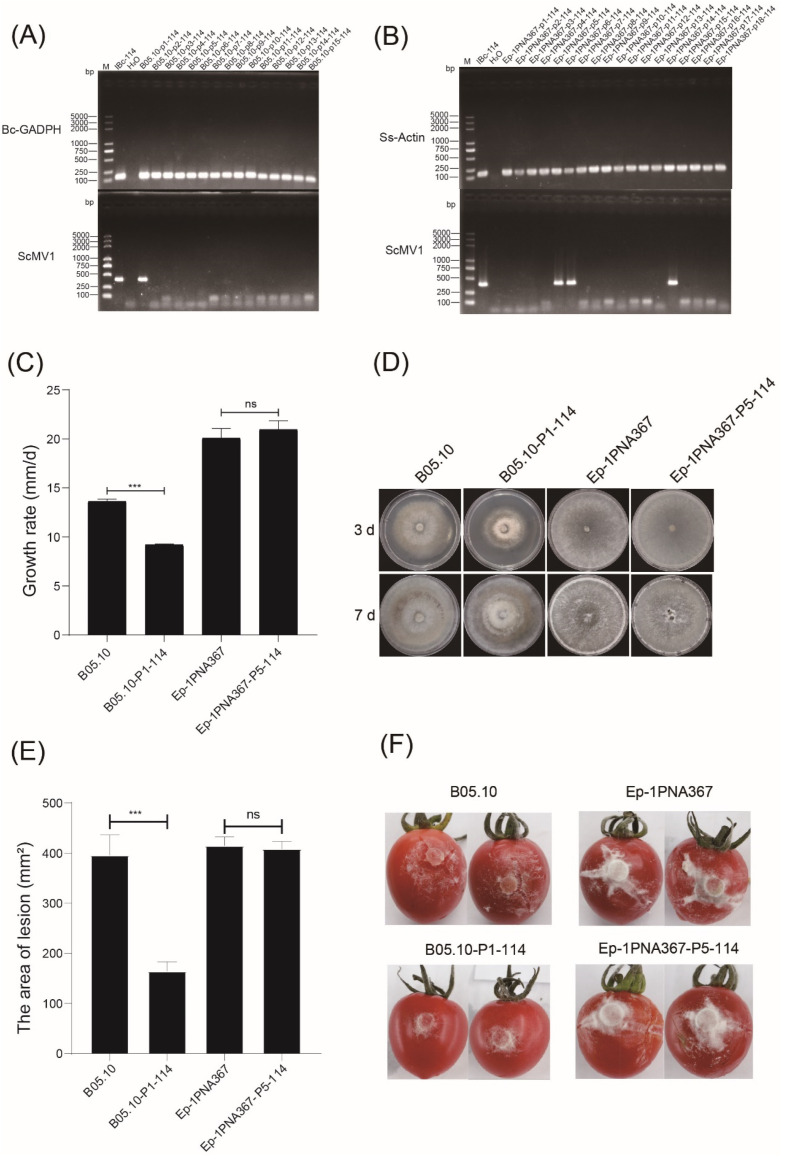
Viruses and biological characteristics of *B. cinerea* and *S. sclerotiorum* transfectants. (**A**) Virus detection was conducted after transfecting *B. cinerea* B05.10 with the total RNA from strain IBc-114. (**B**) Virus detection was conducted after transfecting *S. sclerotiorum* Ep-1PNA367 with the total RNA from strain IBc-114. (**C**) The growth rate of strains B05.10, B05.10-P1-114, Ep-1PNA367 and Ep-1PNA367-P5-114 on PDA at 20 °C. (**D**) The colony morphology of strains B05.10, B05.10-P1-114, Ep-1PNA367 and Ep-1PNA367-P5-114 on PDA at 20 °C. (**E**) The area of lesions caused by strains B05.10, B05.10-P1-114, Ep-1PNA367 and Ep-1PNA367-P5-114 on detached tomato fruits at 4 dpi. The data are presented as means ± SD (*n* = 5). “***” indicates significant difference (*p* < 0.001), while “ns” indicates no significant difference. (**F**) The lesions of strains B05.10, B05.10-P1-114, Ep-1PNA367 and Ep-1PNA367-P5-114 on the detached tomato fruits at 4 dpi.

To further confirm whether BcMV9/IBc-114 infects *B. cinerea* and *S. sclerotiorum*, the total RNA of strain IBc-114 was used to transfect *B. cinerea* strain B05.10 and *S. sclerotiorum* strain Ep-1PNA367. The specific PCR product corresponding to RNA of BcMV9/IBc-114 was detected in *B. cinerea* strain B05.10 as well as in *S. sclerotiorum* strain Ep-1PNA367 (Figure 10A,B). The result suggested that BcMV9/IBc-114 replicated successfully in *B. cinerea* and *S. sclerotiorum*, while ScRV1 did not.

To assess the impact of the virus BcMV9/IBc-114 on its fungal host, we evaluated the biological characteristics of strains B05.10-P1-114 and A367-P5-114. This evaluation was based on growth rate, colony morphology, and virulence on detached tomato fruits. The growth rate of strain B05.10-P1-114 (9.2 ± 0.1 mm/d) was significantly lower than that of strain B05.10 (13.6 ± 0.2 mm/d) (Figure 10C); however, strain B05.10-P1-114 exhibited a colony morphology similar to that of the control strain B05.10 (Figure 10D). The lesion area of strain B05.10-P1-114 was 164.4 ± 18.63 mm^2^, which was significantly smaller than that of strain B05.10 (396.0 ± 40.23 mm^2^) at 4 dpi (Figure 10E,F). The growth rate of strain A367-P5-114 (21.0 ± 0.9 mm/d) was not significantly different from that of strain Ep-1PNA367 (20.1 ± 0.99 mm/d) (Figure 10C). Strain A367-P5-114 exhibited a colony morphology similar to that of the control strain Ep-1PNA367 (Figure 10D). The lesion area of strain A367-P5-114 (408.5 ± 14.93 mm^2^) was not significantly different from that of strain Ep-1PNA367 (415.0 ± 17.71 mm^2^) at 4 dpi (Figure 10E,F). These results demonstrate that BcMV9/IBc-114 decreased the growth rate and pathogenicity of strain B05.10 on PDA, but it had no effect on strain Ep-1PNA367. Furthermore, BcMV9/IBc-114 did not significantly affect the colony morphology of either B05.10 or Ep-1PNA367.

## 4. Discussion

In this study, we identified two mycoviruses from saprotrophic fungus *S. commune*: one is the novel +ssRNA virus ScRV1 that is distantly related to benyvirus, and another is BcMV9/IBc-114. ScRV1 may represent a member of the novel viral lineage in the order *Hepelivirales*, while BcMV9/IBc-114 was confirmed to replicate both in ascomycetous fungi *B. cinerea* and *S. sclerotiorum.*

ScRV1 is significantly different from viruses in the family *Benyviridae*. The family *Benyviridae* established by ICTV has a positive single-stranded and multi-segment genome and includes only one genus *Benyvirus* (https://ictv.global/report/chapter/benyviridae/benyviridae, accessed on 6 August 2024). All members of the genus *Benyvirus* are capable of infecting plants [37]. Members of *Benyvirus* reported in plants have four to five genome segments ranging from 1.3 to 6.7 kb in size, which form unenveloped, rod-like virions ranging from 85 to 390 nm in length and about 20 nm in diameter [37]. Beet necrotic yellow vein virus (BNYVV) is the typical member of the genus *Benyvirus*, which has four to five genome segments of 6.7, 4.6, 1.8, 1.4 and 1.3 kb in length [38,39]. RNA1 of BNYVV is the only one with a large ORF that encodes the replication-associated protein; RNA2 of BNYVV contains six ORFs, the first of which encodes a coat protein of 21–23 kDa. Here, the genome of ScRV1 is a monopartite +ss RNA genome with a size of 7370 nt, and this genome encodes two proteins, one is RdRP, and another is a coat protein. Although the RdRP of ScRV1 is somewhat phylogenetically related to those of the viruses in *Benyviridae* and distantly related to those of the viruses in *Hepeviridae*, the coat protein of ScRV1 is related to those of the viruses in *Alphatetraviridae*. Furthermore, ScRV1 has filamentous particles 20 ± 2.1 nm in diameter and 1000 ± 94.2 nm in length; the particle shape and size are different from those of benyvirus. Based on the genome organization and the phylogenetic analysis of both RdRp and the coat protein of ScRV1, ScRV1 may represent a member in a novel virus family which is related to *Benyviridae*.

In recent years, some mycoviruses in which the RdRP phylogenetically is related to benyviruses were also found in the fungi of phyla Basidiomycota, Ascomycota, and Zoopagomycota; they often were called beny-like viruses. Among them, 11 were identified from basidiomycetous fungi *Agricus bisporus* [40], *Lentinula edodes* [41], *Rhizoctonia* spp. [42], and *Sclerotium rolfsii* (MH766487), and two were identified from ascomycetous fungi, *Monilinia* spp. [43]. With the exception of Lentinula edodes ssRNA mycovirus and Agaricus bisporus virus 13, the remaining viruses form a phylogenetic cluster with ScRV1 and differ from viruses of *Benyviridae* and other families. Thus, we propose here a novel family Mycobenyviridae to adopt ScRV1 and other beny-like viruses. Interestingly, five beny-like viruses have been identified from insect *Bemisia tabaci*; these insect viruses are most closely phylogenetically related to Entomophthora benyvirus E that infects entomopathogenic fungus *Entomophthora* sp., suggesting that viruses of Mycobenviridae may also jump into insects by entomopathogenic fungi. Alternatively, insects may be infected with fungi that are already infected with viruses. Furthermore, a beny-like virus, Bramycfau virus 1 (OL569485), was identified by using the metagenome-assembled genome from ant. This virus is very close to Agaricus bisporus virus 8 that infects mushroom. Since mushrooms are a favorite food for ants, it is suggested that Bramycfau virus 1 is also associated with fungi.

The genome diversity of members in the proposed family Mycobenviridae is striking (Figure S1). The genome size in the family has a range of 5280 nt (Monilinia fructicola beny-like virus 1) to 12,475 nt (Rhizoctonia cerealis beny-like virus isolate RcBeLV-10125-1) in spite of some viral genomes perhaps not being completely sequenced or confirmed. Viruses with a large genome size (over 10 kb) are identified from *Rhizoctonia* spp., while the genome size of other viruses is around 7 kb. The viruses with a large genome size in other families also often found in *Rhizoctonia* spp. [44]; this may be a common characteristic of viruses that infect *Rhzoctonia* spp. Some viruses, such as ScRV1, Agaricus bisporus virus 8, Monilinia fructicola benyvirus 1 and Monilinia benyvirus C have two or three large ORFs, while others may have only one ORF encoding RdRP. Of course, the viral genomes of these viruses with only one RNA segment or with only one ORF need to be further confirmed.

The family *Mitoviridae* features capsid-less viruses with a non-segmented positive-sense single-stranded RNA genome and accommodates four genera: *Duamitovirus*, *Kvaramitovirus*, *Triamitovirus* and *Unuamitovirus* (https://talk.ictvonline.org/, accessed on 6 August 2024) [45]. A large number of mitoviruses have been identified in a variety of fungi [46,47,48,49,50,51,52]. Mitoviruses are common in fungi of Ascomycota; for example, 10 mitoviruses were identified in *B. cinerea* [16,53], 12 were detected in *S. sclerotiorum* [27,54,55,56,57], 9 were reported in the genus *Fusarium* spp. [47,58,59,60,61], 3 were found in the genus *Alternaria* spp. [62,63,64], 5 were found in *Cryphonectria* spp. [48], 2 were found in *Gremmeniella abietina* [65,66], 6 were found in *Ophiostoma novo-ulmi* [67,68], 2 were found in *Botryosphaeria dothidea* [69,70], and 1 was found in *Buergenerula spartinae* [71]. Relatively, only a few mitoviruses have been identified from basidiomycetous fungi, such as in *Agaricus bisporus*, *Heterobasidion* spp., *Lentinula edodes*, *Puccinia striiformis* and *Rhizoctonia* spp. [40,72]. In this study, for the first time, a mitovirus, BcMV9/IBc-114, was discovered in *S. commune*; interestingly, this mitovirus also was previously found in *B. cinerea*.

A persistent question about mycoviruses is how they transmit in fungal host populations in nature, especially transmission among different fungal species. Mycoviruses could transmit via hyphal anastomosis among vegetative-compatible individuals of the host, and vegetative incompatibility is a major barrier to the transmission of mycoviruses in host individuals [6]. More mechanisms for mycovirus transmission among host individuals have been discovered; for example, some mycoviruses could suppress the fungal vegetative incompatibility reaction to facilitate transmission and help co-infected mycoviruses to transmit in host individuals [73]. Even a plant being attacked by fungal pathogens can produce proline to weaken the fungal vegetative incompatibility reaction and help mycovirus transmission [74]. A few mycoviruses, such as SsHADV-1, can use mycophagous insects as vectors for transmission [75]. The fact is that more and more mycoviruses have been identified from different fungal species: for example, mycoviruses with high aa sequence identity were found in both *S. nivalis* and *S. sclerotiorum* [76], in both *B. cinerea* and *S. sclerotiorum* [15,16], in both *B. porri* and *S. sclerotiorum* [14,77], and in both *G. abietina* and *S. nivalis* [78]. The same species of mitoviruses also were found in different fungal species; for example, a strain of Botrytis cinerea mitovirus 1 was identified in *Ophiostoma novo-ulmi*, which is a destructive pathogen that infects elm [67]. Some mycoviruses can carry out cross-species transmission: for example, Cryphonectria naterciae fusagravirus 1 could infect persistently *C. naterciae* and other *Cryphonectria* species by co-culturing [79]. Leptosphaeria biglobosa botybirnavirus 1 (LbBV1) can cross-class transmit from *L. biglobosa* to *B. cinerea* in nature [17]. In this study, we isolated BcMV9, which was first identified in an ascomycetous fungus *B. cinerea*, from *S. commune*, which is a basidiomycetous fungus. This suggests that BcMV9/IBc-114 could be transmitted across phyla between ascomycetous fungi and basidiomycetous fungi. We further confirmed that BcMV9/IBc-114 really could replicate in ascomycetous fungi *B. cinerea* and *S. sclerotiorum* by transfection assay. It is not likely that the interspecies transmission of mycovirus is dependent on hyphal anastomosis because it cannot occur between different fungal species; the mechanism for mycovirus transmission between different fungal species is still unknown. In this study, strain IBc-114 was sampled from a gray mold lesion on cucumber. Initially, it was regarded as an abnormal (conidia-less) strain of *B. cinerea*. However, it was later identified as *S. commune*. Strain IBc-114 (*S. commune*) and *B. cinerea* share the same ecological niche. This indicates that mycoviruses may be capable of cross-species transmission among fungi inhabiting the same niche. This suggests that BcMV9/IBc-114 may act as a competitive advantage for *S. commune* against *B. cinerea.* Considering some small RNA was found to be capable of cross-kingdom transmission [80,81], viral RNA when released into ambient air by a fungal donor may be able to successfully infect other fungal species [82].

Furthermore, we found that ScRV1, but not BcMV9/IBc-114, could inhibit the hyphal growth of *S. commune*. However, hyphal growth and pathogenicity are suppressed in *B. cinerea* infected with BcMV9/IBc-114. This suggests that BcMV9/IBc-114 may be a secret weapon for *S. commune* to compete with *B. cinerea*. BcMV9/IBc-114 has little impact on *S. commune* and *S. sclerotiorum*, but it has a significant effect on *B. cinerea*, which suggests that the impact of the virus on the host is not solely determined by the virus itself; it may be a combination of the virus and the host. In addition, screening mycoviruses from saprophytic fungi surrounding pathogenic fungi to control disease is also a potential new approach, and the potential use of BcMV9/IBc-114 as a biocontrol agent to control gray mold deserves further investigation.

## Figures and Tables

**Table 1:** Homology analysis of the hidden Markov model (HMM) of the protein encoded by ORF2 of ScRV1.

Hit	Probability (%)	Identities (%)	E-Value	*p*-Value	Query HMM	Template HMM
Capsid protein [Hepatitis E virus]	97.8	16	4.3 × 10^−5^	4.5 × 10^−10^	15–152 (242)	80–258 (660)
Capsid protein [Hepatitis E virus genotype 2]	97.48	17	0.0002	2 × 10^−9^	13–152 (242)	78–258 (659)
Capsid protein [Hepatitis E virus type 4]	97.43	17	0.00034	3.5 × 10^−9^	35–152 (242)	2–148 (504)
Capsid protein [Avian hepatitis E virus]	97.14	19	0.0011	1.2 × 10^−8^	22–152 (242)	40–203 (606)
Capsid readthrough protein [Beet necrotic yellow vein virus]	93.80	17	0.011	1.2 × 10^−7^	13–214 (242)	14–174 (690)
Ilarvirus coat protein [Prunus necrotic ringspot virus]	83.72	14	2.9	3 × 10^−5^	1–242	1–205 (208)

## Data Availability

Data is contained within the article or Supplementary Material.

## Supplementary Materials

The following supporting information can be downloaded at https://www.mdpi.com/article/10.3390/v16111767/s1, Table S1: The information of primers used in this study; Table S2: The fungal information selected for multiple sequence alignment analysis and phylogenetic analysis in this study; Table S3: All strains used in this study and the viruses they carry; Supplementary Figure S1: Genomic structures of viruses of the proposed family Mycobenyviridae. CP: coat protein; HP: Hypothetical protein; RdRp: RNA-dependent RNA polymerase.
