# Supplementary material for: A Mycovirus Representing a Novel Lineage and a Mitovirus of Botrytis cinerea Co-Infect a Basidiomycetous Fungus, Schizophyllum commune
Source: Viruses. 2024 Nov 13;16(11):1767. doi: 10.3390/v16111767 (PMC11598958; doi:10.3390/v16111767)
Supplement: Supplementary file 1 [file viruses-16-01767-s001.zip › Table S1 The information of primers used in this study.pdf]

Table S1. The information of primers used in this study.

**Sequence/Gene name**	**Primer name**	**Primer Sequence**
Actin	SC-ACTIN-F1	CGGGTATCCACGAGACGA
	SC-ACTIN-R1	ACTTGCGGTGGACGATGC
BC-1_Contig348	ScRV1-F	GACTTTCTGGGCACTTGGGA
	ScRV1-R	ATAGACCGTGTCGTAGTCCAAGC
BC-3_Contig60	ScMV1-F	TGGACAACCCGATAAGTGTATGAAA
	ScMV1-R	ATTTGCAGAGGAAGTCTATCT
ScRV1	ScRV1-F3	TAAGGAAGCCACCTGGAAGAA
	ScRV1-R3	CCGACACAACGGCGAGTAAA
BcMV9/IBc-114	ScMV1-F	TATGATTACATCCCAAAAGGCGA
	ScMV1-R	GCTGATTTTCTACTATTCGGACCA
ITS	ITS1-F	AAAAAATGTAACAAGGTTTCCGTAG
	ITS4-R	TATGATATGCTTAAGTTCAGCGGGT
Calmodulin	CAL-F	ACCGCAACCACCAACTCCTTA
	CAL-R	ATAACGAAGCAAGAAGGACGATTAC
Ras	RAS1-F	GAGGCTACTGGGATGATTGCG
	RAS1-R	CGCGTGCAGGTAAGATAGTAAGAG
Beta tubulin	TUB-F	TCCGCTCTCACAAGTCATGTAAGTC
	TUB-R	CGAAGAGCAGAAGTCAACCC
