# Supplementary material for: A Mycovirus Representing a Novel Lineage and a Mitovirus of Botrytis cinerea Co-Infect a Basidiomycetous Fungus, Schizophyllum commune
Source: Viruses. 2024 Nov 13;16(11):1767. doi: 10.3390/v16111767 (PMC11598958; doi:10.3390/v16111767)
Supplement: Supplementary file 1 [file viruses-16-01767-s001.zip › Table S2 The fungal information was selected for multiple sequence alignment analysis and phylogenetic analysis in this study..pdf]

Table S2 The fungal information selected for multiple sequence alignment analysis and phylogenetic analysis in this study.

**Fungal species name**	**Associated CBS strains**
Schizophyllum commune	CBS 103.20
S. commune	CBS 109296
S. commune	CBS 124811
S. commune	CBS 249.69
S. commune	CBS 405.96
S. commune	CBS 579.83
S. commune	CBS 342.58
S. commune	CBS 227.57
S. commune	CBS 340.81
S. fasciatum	CBS 267.60
Auriculariopsis ampla	CBS 285.88
A. ampla	CBS 182.83
Verticillium fungicola	CBS 133.97
Botrytis cinerea	CBS:261.71
B. cinerea	CBS:179.71
B. cinerea	CBS:126.58
