# Supplementary material for: A Mycovirus Representing a Novel Lineage and a Mitovirus of Botrytis cinerea Co-Infect a Basidiomycetous Fungus, Schizophyllum commune
Source: Viruses. 2024 Nov 13;16(11):1767. doi: 10.3390/v16111767 (PMC11598958; doi:10.3390/v16111767)
Supplement: Supplementary file 1 [file viruses-16-01767-s001.zip › Table S3 All strains used in this study and the viruses they carry.pdf]

Table S3 All strains used in this study and the viruses they carry.

strains name	Viral name
IBc-114	ScRV1, BcMV9/IBc-114
IBc-114-46	ScRV1
IBc-114-39	/
S. sclerotiorum strain Ep-1PNA367	/
B. cinerea strain B05.10	/
B05.10-p1-114	BcMV9/IBc-114
B05.10-p2 -114	/
B05.10-p3 -114	/
B05.10-p4 -114	/
B05.10-p5 -114	/
B05.10-p6 -114	/
B05.10-p7 -114	/
B05.10-p8 -114	/
B05.10-p9 -114	/
B05.10-p10 -114	/
B05.10-p11 -114	/
B05.10-p12 -114	/
B05.10-p13 -114	/
B05.10-p14 -114	/
B05.10-p15 -114	/
Ep-1PNA367-p1-114	/
Ep-1PNA367-p2-114	/
Ep-1PNA367-p3-114	/
Ep-1PNA367-p4-114	/
Ep-1PNA367-p5-114	BcMV9/IBc-114
Ep-1PNA367-p6-114	BcMV9/IBc-114
Ep-1PNA367-p7-114	/
Ep-1PNA367-p8-114	/
Ep-1PNA367-p9-114	/
Ep-1PNA367-p10-114	/
Ep-1PNA367-p11-114	/
Ep-1PNA367-p12-114	/
Ep-1PNA367-p13-114	/
Ep-1PNA367-p14-114	BcMV9/IBc-114
Ep-1PNA367-p15-114	/
Ep-1PNA367-p16-114	/
Ep-1PNA367-p17-114	/
Ep-1PNA367-p18-114	/

"/" represents that there is no virus in the strain.
