# Supplementary figures and images for: A Mycovirus Representing a Novel Lineage and a Mitovirus of Botrytis cinerea Co-Infect a Basidiomycetous Fungus, Schizophyllum commune
Source: Viruses. 2024 Nov 13;16(11):1767. doi: 10.3390/v16111767 (PMC11598958; doi:10.3390/v16111767)

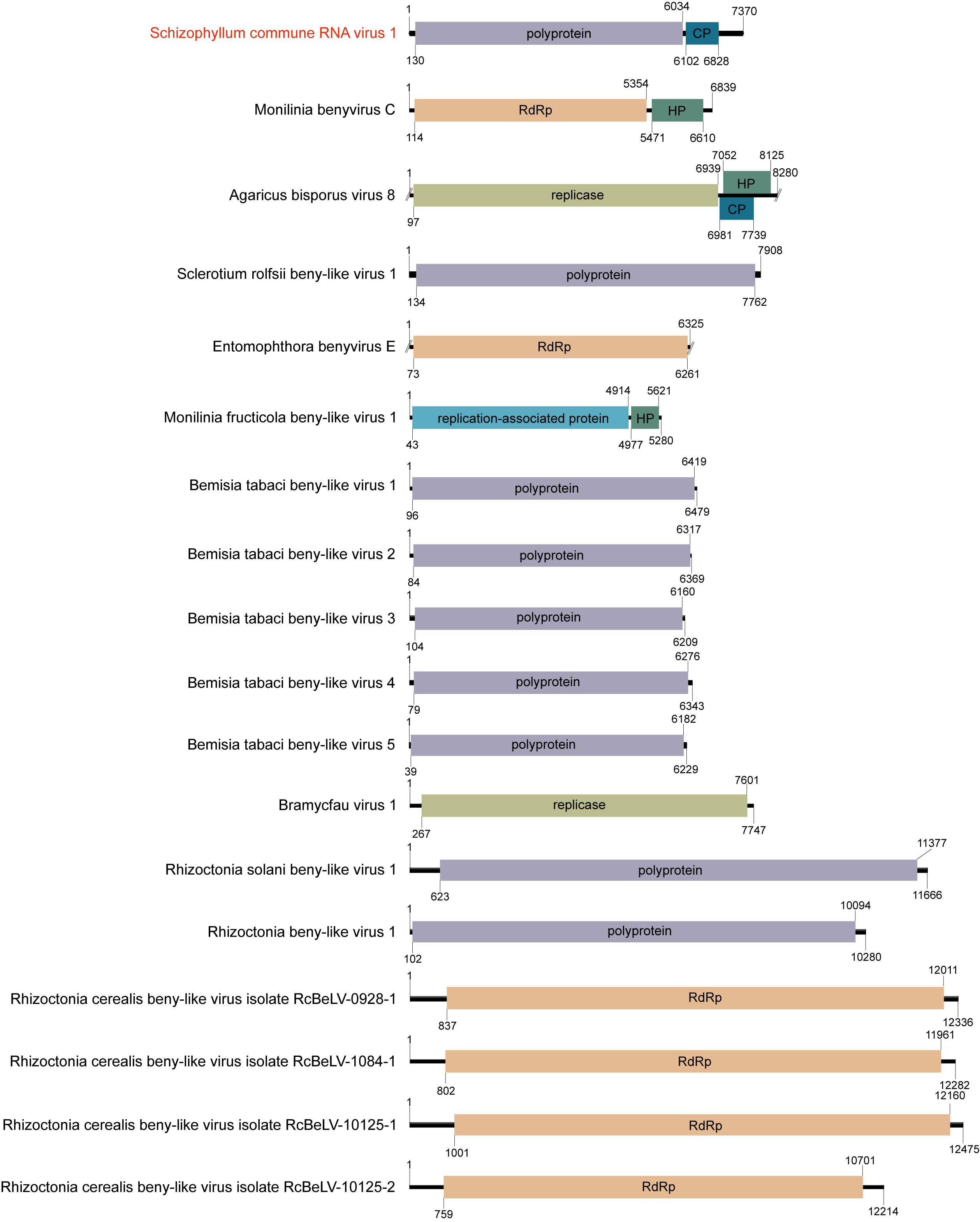

Supplement: Supplementary file 1 [file viruses-16-01767-s001.zip › Supplementary Figure S1.tif]
